# The prevalence of metabolic syndrome amongst patients with severe mental illness in the community in Hong Kong – a cross sectional study

**DOI:** 10.1186/1471-244X-13-87

**Published:** 2013-03-18

**Authors:** Daniel T Bressington, Jolene Mui, Eric F C Cheung, Joel Petch, Allan B Clark, Richard Gray

**Affiliations:** 1 The Department of Health, Well-being and the Family, Canterbury Christ Church University, North Holmes Road, Canterbury, Kent, CT1 1QU, UK; 2 Community Psychiatric Service, Castle Peak Hospital, 15 Tsing Chung Koon Road, Tuen Mun, New Territories, Hong Kong; 3 Castle Peak Hospital, 15 Tsing Chung Koon Road, Tuen Mun, New Territories, Hong Kong; 4 Norwich Medical School, University of East Anglia, Norwich, NR4 7TJ, UK; 5 University of the West of England, Frenchay Campus, Coldharbour Lane, Bristol, BS16 1QY, UK

**Keywords:** Severe mental illness, Metabolic syndrome, Health behaviours, Physical health screening

## Abstract

**Background:**

Patients with severe mental illness are at increased risk of developing metabolic disorders. The risk of metabolic syndrome in the Hong Kong general population is lower than that observed in western countries; however the prevalence of metabolic syndrome in patients with severe mental illness in Hong Kong is unknown.

**Method:**

This cross-sectional study aimed to estimate the prevalence of metabolic syndrome in patients with severe mental illness in Hong Kong and to identify the relationships between metabolic syndrome and socio-demographic, clinical and lifestyle factors.

**Results:**

A total of 139 patients with a diagnosis of severe mental illness participated in the study. The unadjusted prevalence of metabolic syndrome was 35%. The relative risk of metabolic syndrome in comparison with the general Hong Kong population was 2.008 (95% CI 1.59-2.53, p < 0.001). In a logistic regression model sleep disruption and being prescribed first generation antipsychotics were significantly associated with the syndrome, whilst eating less than 3 portions of fruit/vegetables per day and being married were weakly associated.

**Conclusion:**

The results demonstrate that metabolic syndrome is highly prevalent and that physical health inequalities in patients with severe mental illness in Hong Kong are similar to those observed in western countries. The results provide sufficient evidence to support the need for intervention studies in this setting and reinforce the requirement to conduct regular physical health checks for all patients with severe mental illness.

## Background

The physical health of patients with severe mental illness (SMI) in western societies has been shown to be extremely poor. It is estimated that patients with SMI have a life expectancy up to 25 years less than the general population [1] and that lifespan is worsening over time. Hong Kong has one of the highest life expectancies in the world [2]. To date there have been few empirical studies that provide estimates of whether physical health inequalities for patients in Hong Kong with SMI are similar to those observed in western countries.

Studies in the UK and USA demonstrate that patients with SMI are more likely to develop cardiovascular disease and have a much higher rate of metabolic disorders than the general population [3]. A UK study [4] involving almost 800 SMI patients screened for physical health issues identified that 66% of patients had a Body Mass Index of over 25 and that risk factors for cardiovascular disease were very common. Metabolic syndrome (MES) is also commonly seen in patients with SMI; a study involving 689 participants in the USA [5] identified that males with schizophrenia were 138% more likely and females 251% more likely to have MES than the general population. MES is defined by the International Diabetes Federation as a collection of clinical indicators; including central obesity (measurable by waist circumference), hypertension and altered lipid levels, amongst other clinical features. MES is reported to be a key risk factor for cardiovascular disease and diabetes mellitus both of which impact heavily upon life quality and expectancy [6].

To our knowledge the prevalence of metabolic syndrome in patients with SMI in Hong Kong has not been reported in the literature to date. However previous research has explored the prevalence in the general population, for example a study involving over 7,000 people estimated that the unadjusted prevalence of MES using the International Diabetes Federation (IDF) criteria was 17.6% [6]. An earlier smaller study [7] using the same IDF criteria estimated a prevalence rate of 7.4% in a Hong Kong working population. Findings reported by Thomas et al [8] reinforce the importance of identifying and treating MES in a Hong Kong setting as the syndrome was associated with an increased risk of cardiovascular disease (hazard ratio 6.39, 95%CI 1.40-29.2) and increased all-cause mortality risk (hazard ratio 2.02, 95% CI 1.02-4.00).

### Study aims

The aims of this study are to:

• Estimate the prevalence of metabolic syndrome in community dwelling patients with SMI in Hong Kong;

• Identify the relationships between metabolic syndrome and socio-demographic, clinical, treatment and lifestyle factors in the study population.

## Methods

### Study design

This exploratory study uses a cross-sectional survey design to estimate the prevalence of MES in the sample population. We report an analysis of baseline data from the initial findings of a larger on-going prospective case series study. In order to ensure that the sample size obtained provided adequate statistical power we performed a power calculation. Based on the assumption that there are 800 patients with SMI registered with the CPN service, with a margin for error of ±10% and a 95% confidence interval, we calculated that we would require a sample size of 86 participants.

### Recruitment and selection of participants

30 Community Psychiatric Nurses (CPNs) working in the clinical setting screened and recruited participants. The study used a convenience sampling approach on a sequential basis, where each CPN asked the first five service users that they were routinely scheduled to visit and who met the inclusion criteria to participate in the study.

#### Inclusion criteria

• Male or female. Aged over 18; with a diagnosis of Severe Mental Illness (SMI) defined by a case-note diagnosis of any psychotic disorder (i.e. schizophrenia, schizoaffective disorder), bipolar affective disorder (type 1 or 2) or psychotic depression.

#### Exclusion criteria

• Any service user who did not have capacity to provide informed consent.

### Ethical considerations

Ethical approval was obtained from the Hong Kong New Territories West Cluster Clinical and Research Ethics Committee. Participants were required to provide written informed consent in order to participate, the participant information sheets clearly stated that declining to take part would not negatively influence their clinical treatment. We chose to ask CPNs to recruit participants and collect data for a number of reasons. As recruitment and data collection was mainly carried out in the home environment we felt that it would be more appropriate for a CPN who knew the service users and their families well to carry out the examinations rather than an independent researcher. We also wanted to ensure that the CPNs would be immediately aware of any physical health concerns identified by the screening and hence recommend or provide clinical interventions as deemed appropriate.

### Data collection

The Health Improvement Profile (HIP) [9] was used as a screening tool to collate data about the physical health state of individuals and their health-related behaviours. The community psychiatric nurses (CPNs) who collected data were trained how to use the HIP and carry out the required physical examinations shortly before the study commenced.

The HIP is a 27 item tool that is gender specific and is designed to be used as both a screening measure and a tool to facilitate interventions aimed to improve physical health and associated health behaviours [9]. Parameters are provided for each item in order that aspects of health and lifestyle can be flagged as red (indicating that an intervention is required) or green (no intervention recommended). The authors conducted literature reviews to establish the “normal” and “at risk” ranges and developed the tool through a series of pilot studies [10]. The utility and acceptability of the HIP as a screening tool was established in an exploratory case series [11]. In this study we analysed the health behaviours that our literature review revealed were most likely to be associated with metabolic syndrome.

The HIP was originally designed for use in a western population and therefore we modified the parameters of the HIP that relate to obesity to reflect the most recent guidelines for the assessment of MES in an Asian population. A BMI of 23 or over was used to represent being overweight and a waist circumference of 90 cm for males was used to determine central obesity. In line with recommendations from the International Diabetes Federation [12] we used the following criteria to determine MES in study participants: (Table 1).

**Table 1:** International diabetes federation criteria for metabolic syndrome in an Asian/Chinese population

**Central obesity** - defined by with ethnicity specific waist circumference of ≥ 80 cm (females) and ≥ 90 cm (males) plus any two of the following four factors:	
**Raised triglycerides**	≥ 150 mg/dL (1.7 mmol/L)
	or specific treatment for this lipid abnormality
**Reduced HDL cholesterol**	< 40 mg/dL (1.03 mmol/L) in males
	< 50 mg/dL (1.29 mmol/L) in females
	or specific treatment for this lipid abnormality
**Raised blood pressure**	Systolic BP ≥ 130 or diastolic BP ≥ 85 mm Hg or treatment of previously diagnosed hypertension
**Raised fasting plasma glucose**	(FPG) ≥ 100 mg/dL (5.6 mmol/L),
	or previously diagnosed type 2 diabetes

(adapted from IDF; 2006) [12].

In addition to the HIP data we also recorded a range of demographic and clinical characteristics: age, gender, education, marital status, diagnosis, duration of illness, employment status and prescribed medication. CPNs carried out basic physical examinations and data from blood tests were obtained from the servicer users’ most recent outpatient medical records. All plasma glucose levels were obtained whilst participants were fasting.

### Data analysis

Data were analysed using SPSS (version 17). We used the presence of MES as the dependent variable and conducted Chi-square tests or Fisher’s exact tests to identify differences between demographic, clinical, treatment related and health behaviour variables in those with and without MES (in order that potential associations with MES could be highlighted). We also conducted independent samples T-tests for the continuous variables to determine differences in mean values for patients with and without MES. In a secondary analysis Chi-square tests were used to determine differences in cardiovascular risk factors according to gender.

In order to identify the independent risk factors associated with metabolic syndrome, the variables that are not part of the criteria for the syndrome were selected from a forward selection criterion by sequentially including variables in the logistic regression model until none were significant at the 0.2 level. We included variables in the final regression model that were significantly associated at the p < 0.05 level and also those weakly associated at the p < 0.10 level. In order to calculate the relative risk of developing MES in our sample we used data reported by Ko and Tang [6] for the prevalence in the general Hong Kong population.

## Results

A total of 139 participants met the analysis criteria and had sufficient data recorded to estimate the prevalence of MES. Data were collected during February 2012.

### Demographic and clinical characteristics of study participants

Table 2 details the demographic and illness characteristics of study participants.

**Table 2:** Demographic and clinical characteristics of participants

**Demographic**	**Number (%)**
**Gender**	
Male	62 (45)
Female	77 (55)
**Marital Status**	
Single	93 (67)
Married	36 (26)
Widowed	8 (6)
Unknown	2 (1)
**Educational Level**	
None	6 (4)
Primary	49 (35)
Secondary	80 (58)
University/Post Graduate	4 (3)
**Diagnosis**	
Schizophrenia and related disorders	97 (70)
Bipolar Affective Disorder (BPAD)	14 (10)
Psychotic depression	19 (14)
Other (i.e. schizoaffective disorder; non-specified psychosis)	8 (6)
Unknown	1 (<1)
**Employment Status**	
Unemployed	68 (49)
Employed	33 (24)
Homemaker	32 (23)
Retired/other	4 (3)
Unknown	2 (1)

The mean age of participants was 47 years (sd = 11.1, range 18-70). All patients were Hong Kong Chinese, the majority were female and two-thirds were single. Around half of participants were unemployed. In terms of educational level; a third had completed primary school, over half had completed secondary education and very few (3%) had further/higher qualifications.

In terms of illness/treatment characteristics; almost three quarters of patients had a diagnosis of schizophrenia and related disorders, 19 (14%) had psychotic depression and one in ten were diagnosed with bipolar affective disorder. On average they had been in contact with psychiatric services for 14 (sd = 10.8) years.

Medications prescribed, individual prevalence of MES in each medication and their statistical association with MES are detailed in Table 3.

**Table 3:** Prescribed medication and association with MES

**Prescribed first generation antipsychotics**	**Number (%)**	**Number of patients with MES (%)**	***χ***^**2**^**, P-value**
Yes	51 (37)	25 (49)	*χ*^2^ = 6.69, p = 0.01
No	88 (63)	24 (27)	
**Prescribed Second Generation Antipsychotics**			
Yes	81 (58)	26 (32)	NS
No	58 (42)	23 (39)	
**Prescribed Clozapine**			
Yes	16 (12)	5 (31)	NS
No	123 (89)	44 (36)	
**Prescribed Long Acting Antipsychotic Injection**			
Yes	33 (24)	14 (42)	NS
No	106 (76)	35 (33)	
**Prescribed an antihypertensive**			
Yes	32 (23)	18 (56)	*χ*^2^ = 8.03, p < 0.01
No	107 (77)	31 (29)	
**Prescribed a statin**			
Yes	6 (4)	6 (100)	*χ*^2^ = 11.52, p < 0.01
No	133 (96)	43 (32)	
**Prescribed diabetes treatment**			
Yes	16 (12)	12 (75)	*χ*^2^ = 12.52, p < 0.01
No	123 (89)	37 (30)	

P for association between prescribed medications and metabolic syndrome, tested by *χ*^2^ test.

*NS* = Not significant.

Over half of participants were prescribed second generation (atypical) antipsychotic medication, and just over a third was prescribed first generation (typical) antipsychotics. Around one in ten patients was being treated with clozapine. Around a quarter of service users were prescribed an antihypertensive, only 16 (12%) were being offered diabetes treatments and very few (n = 6, 4%) were prescribed a statin.

### Prevalence of MES and other cardiovascular risk factors

The prevalence, relative risk of MES and cardiovascular risk indicators split according to gender are shown in Table 4.

**Table 4:** Prevalence of MES (with relative risk) and indicators of cardiovascular risk (split according to gender)

	**Male n = 62, (%)**	**Female n = 77, (%)**	**Total n, (%)**	***χ***^**2**^, P-value*
Metabolic Syndrome present	19 (31)	30 (39)	49 (35)	NS
Relative Risk of Metabolic Syndrome (95% CI, P value)**	1.737 (1.18-2.54, p < 0.01)	2.230 (1.67-2.97, p < 0.01)	2.008 (1.59-2.53, p < 0.01)	
BMI overweight- Hong Kong criterion (≥23)	48 (77)	63 (82)	111 (80)	NS
BMI overweight- International criterion (≥25)	34 (55)	45 (58)	79 (57)	NS
Waist Circumference (males ≥90 cm, females ≥ 80 cm)	20 (32)	47 (61)	67 (48)	*χ*^2^ = 11.40, p < 0.01
Hypertension – Blood pressure ≥ 140/90	12 (19)	21 (27)	33 (24)	NS
Hypercholesterolemia – Total Cholesterol ≥6.2 mmol/L	15 (24)	16 (21)	31 (22)	NS
Raised LDL cholesterol – LDL ≥ 4.1 mmol/L	11 (18)	12 (16)	23 (16)	NS
Reduced HDL cholesterol – HDL <1.00 mmol/L (males), <1.30 mmol/L (females)	13 (21)	19 (25)	32 (23)	NS
Raised fasting plasma glucose - ≥ 7.00 mmol/L	8 (13)	9 (12)	17 (12)	NS
Raised triglycerides - ≥2.2 mmol/L	17 (27)	17 (22)	34 (25)	NS

*P for significant differences in cardiovascular risk according to gender, tested by *χ*^2^ test.

*NS* = not statistically significant.

** Data reported by Ko and Tang [6] for the prevalence of MES in the general Hong Kong population was used to calculate the relative risk in this sample.

The unadjusted prevalence of MES in the entire sample was just over a third (35%, n = 49). When data are split according to gender; 31% (n = 19) of males and 39% (30) females met the diagnostic criteria for MES. The vast majority of patients (80%, n = 111) had a BMI of 23 or over.

Around a quarter of participants (24%, n = 33) were reported to be hypertensive, raised triglycerides were observed in 34 (25%) of service users and 32 (23%) were found to have reduced levels of HDL cholesterol. Raised fasting plasma glucose levels were detected in 17 (12%) of patients.

In comparison with males a higher percentage of women were observed to be overweight, however Chi-square tests demonstrate that the only cardiovascular risk factor that is significantly more likely in women is a waist circumference over recommended limits (*χ*^2^ = 11.40,p = 0.001).

### Health behaviours of study participants

The self-reported health behaviours of participants and associations with MES are shown in Table 5. Very few participants used cannabis (n = 3, 2%) or drank more than 4 units of alcohol per day (n = 7, 5%), similarly few participants consumed more than 600 mg of caffeine each day (n = 8, 6%) and 38 (27%) smoked tobacco. The majority of patients (n = 86, 62%) exercised for less than recommended levels (30 minutes per day five times per week) and around a fifth ate more than 3 portions of fat per day (n = 27, 19%) and 26 (19%) consumed less than three portions of fruit/vegetables per day.

**Table 5:** Health behaviours of participants

**Health behaviour**	**Total n, (%)**	***χ***^**2**^**, P-value**
Smoking status (smoker/passive smoker)	38, (27)	NS
Alcohol intake (>4 units daily)	7, (5)	NS
Exercise (< 30 minutes a day for 5 days per week)	86, (62)	NS
Diet fruit and vegetables (≤2 daily portions)	26, (19)	NS
Diet Fats (≥ 3 portions per day)	27, (19)	NS
Fluid Intake (<1 L or >3 L per day)	9, (7)	NS
Caffeine Intake (≥600 mg/day)	8, (6)	NS
Disrupted sleep (>8 h or <3 h)	38, (27)	*χ*^2^ = 4.64,p < 0.05
Cannabis use (occasional or regular)	3, (2)	NS

P for association between health behaviour and metabolic syndrome, tested by *χ*^2^ test.

*NS* = Not statistically significant.

### Associations with MES

#### Demographic and clinical characteristics

None of the demographic or clinical characteristics were found to be significantly associated with MES in this group of patients. However, independent sample T-tests demonstrate that there is a trend towards the mean duration of illness being longer in the group of patients with metabolic syndrome (t = 1.94, df = 137, p = 0.054).

#### Treatment-related variables

There were significant relationships observed between MES and medical treatments for associated issues; diabetes treatments (*χ*^2^ = 12.52, p < .001), statins (*χ*^2^ = 11.52, p < .001) and antihypertensives (*χ*^2^ = 8.03, p < .01) were all strongly associated with MES. As these variables constitute elements of the criteria for MES this result was expected and therefore the associations are not particularly clinically relevant.

In terms of medicines prescribed for mental health, only 1st generation antipsychotics were significantly associated with MES (*χ*^2^ = 6.69, p < .01). Surprisingly there was no relationship observed between numbers of antipsychotics prescribed, clozapine or atypical antipsychotics and MES.

#### Health behaviour related variables

Of all the health-behaviour related variables only sleep disruption (<3 hours or >8 hours per night) was associated with MES (*χ*^2^ = 4.64, p = 0.031). A secondary analysis further demonstrated that when all the health behaviour variables are grouped together as one risk factor they were not significantly associated with MES.

#### Independent related variables

Table 6 shows the adjusted odds ratios for independent risk factor variables which were significant at the 0.2 level in the regression model. In the logistic regression model sleep disruption (OR 3.57 (95% CI 1.45, 8.79), p = 0.006), being prescribed first generation antipsychotics (OR 3.83 (95% CI 1.63, 9.04), p = 0.002) were significant risk factors for MES. The model also shows that eating less than 3 portions of fruit/vegetables per day (OR 2.19 (95% CI 0.86,5.59), p = 0.099) and being married (OR 0.45 (95% CI 0.19,1.09), p = 0.077) were weakly significant risk factors.

**Table 6:** Adjusted odds ratios for the presence of metabolic syndrome

**Factor**	**Odds ratio (95% CI)**	**p-value**
First generation antipsychotics	3.83 (1.63,9.04)	0.002
Disrupted sleep (>8 h or <3 h)	3.57 (1.45,8.79)	0.006
Marital status (single/widowed/divorced vs. married)	0.45 (0.19,1.09)	0.077
Diet fruit and vegetables	2.19 (0.86,5.59)	0.099

## Discussion

Metabolic syndrome is highly prevalent in this cohort of patients; 35% of the participants (31% of men and 39% of women) met the IDF criterion. Previous studies show that the prevalence of MES in patients with SMI differs in accordance with the population being studied. Research carried out in the USA has estimated the prevalence of MES in SMI to be between 28.7% [13] and 60% [14]. A similarly high prevalence rate of 54% was reported in an Australian study [15]. Whilst a Canadian study calculated a prevalence rate of 44.7% [16] and a Finnish project [17] estimated 37.1%. Studies that explore Asian populations tend to report lower rates of MES in SMI than western countries, for example Littrell et al [18] report that 22% of patients with schizophrenia in Taiwan had MES, whilst in Thailand Srissurapanont et al [19] estimated a prevalence rate of 20%. Although direct comparisons with other countries are complicated by the differing prevalence rates in the general populations, our results suggest that the rates of MES in patients with SMI in Hong Kong are likely to be lower than those observed in western populations and higher than in some other Asian countries.

It is perhaps more clinically relevant to report the increased risk of MES in SMI with the general population (please see Table 4). In the general Hong Kong community the unadjusted prevalence rates of MES have been reported as 17.6% (17.7% of males and 17.5% of females) [6]. As our results indicate that 35% of patients with SMI have MES (31% men and 39% women) the relative risk of metabolic syndrome in comparison with the general Hong Kong population is 2.008 (95% CI 1.59-2.53, p < 0.001). The relative risk for males is 1.737 (95% CI 1.18-2.54, p = 0.005) and for females is 2.230 (95% CI 1.67-2.97, p < 0.001). McEvoy et al., [5] estimate the increased risk of MES in SMI to be 138% for men and 251% for women in the USA. Therefore, although the overall rates of MES in patients with SMI in Hong Kong are lower than in some western countries the increased risk is potentially higher for men, although slightly lower for women, compared to rates reported in the USA.

Despite the high prevalence of MES observed in this study few patients were prescribed medical treatments for dyslipidaemia or diabetes. McEvoy et al., [5] reported similar findings in the USA; significant percentages of SMI patients with these conditions were not receiving treatment. This finding suggests the requirement for enhanced collaborative working between primary care and specialist mental health services, in order that patients can receive prompt appropriate medical treatments once such conditions are identified.

None of the demographic or clinical characteristics were found to be significantly associated with MES in our initial bivariate analysis. However, the relationship between duration of illness and MES was found to be approaching statistical significance. The prevalence of MES in women was higher than in men, but the difference was not significant (*χ*^2^ = 1.04, p = 0.31). These findings are consistent with results reported by John et al., [15] however many other studies identify a clear link between age, gender, duration of psychiatric illness and MES [19,20].

The multivariable logistic regression model highlights that patients in this study who were married were slightly more likely to have developed MES than those who were single/divorced or widowed (OR 0.45 (95% CI 0.19,1.09), p = 0.077). Previous studies that explore the relationship between marital status and MES have reported equivocal results, for example married women in the general population of USA were found to have a lower prevalence of MES than those who were single [21] whilst no such relationships were observed in a study of over 2000 patients in Taiwan [22]. One possible explanation for our result is an inter-relationship between MES, marriage and diet.

Previous authors [23] have proposed that that the unhealthy lifestyles of patients with SMI contribute towards the aetiology of MES. Unexpectedly in the bivariate analysis we did not identify any significant associations between lifestyle variables (other than sleep) and MES in this sample. However, eating less than recommended levels of fruits and vegetables was found to be a weak predictor of MES in the final multivariate regression model. The relationship between poor diet and MES in people with SMI has been reported in many studies i.e. [24]; these previous results and our observations suggest that health promotion approaches aimed at reducing cardiovascular risk need to consider incorporating dietary interventions.

The association between sleep disruption and MES was significant, and the regression model also identified sleep disruption as being a risk factor for the syndrome. This finding has previously been reported by a number of authors, for example; Hall et al [25] conducted a study of over 200 participants in the general population in the USA and concluded that there is an increased risk of developing MES of 45% in people who sleep more or less than 7-8 hours per day. The association between short sleep duration and MES may be explained by the relationship identified between obstructive sleep apnoea and many indicators of MES, for example; obesity, hypertension, diabetes and dyslipidaemia [26]. The evidence relating to an association between a long duration of sleep and MES is less clear; however some authors conclude that risk factors for MES are more common in long sleepers, i.e. type 2 diabetes [25]. The relationship between excess sleep and MES observed in our study may also be explained by the likelihood that some of the more sedating antipsychotic drugs have been observed to be implicated in the development of MES [27], or potentially that those participants with sleep disruption may be more likely to be engaging in a generally more sedentary lifestyle.

First generation (or typical) antipsychotics were observed to have an association with MES and were also significant in our regression model, a surprising finding given that many studies demonstrate that second generation antipsychotics are more likely to be associated with MES than first generation drugs [27]. However, our findings are supported by a number of studies that identify the same association [15,17,28]. One potential explanation for this result is that we did not perform analysis for the association of individual atypical antipsychotic preparations with MES (other than clozapine), and this may be a confounder as some atypical antipsychotics have been reported to be more likely to cause weight gain than others [27]. Nor did we collect data relating to the extent of adherence with antipsychotic medications. It is therefore not possible to draw firm conclusions about the potential impact of these medications on physical health.

In the literature there is continued debate about the likelihood of atypical antipsychotics being involved in the aetiology of MES [15]. The current physical health policy in the clinical setting in which our research was carried out recommends that patients receiving atypical (or second generation) antipsychotics require at least yearly screening for metabolic abnormalities; our results suggest that this should be extended to anyone with a diagnosis of a severe mental illness irrespective of the type of antipsychotic prescribed. Although the mean duration of illness in those with MES was not significantly different to those without MES, the observed trend and previous research suggests that a longer duration of illness may increase the risk of MES. This may also provide a potential explanation of why typical antipsychotics are associated with MES in this study; current treatment guidelines recommend the use of atypicals for patients newly diagnosed and therefore it is likely that those who are taking typical medications have been established on these for some time and subsequently may have a longer duration of illness.

There are a number of study limitations that could have influenced our findings and which also make it difficult to generalise the results to other countries or the wider eastern world. This study has a cross-sectional design, includes a relatively small number of patients with SMI and uses a convenience sampling approach rather than utilising an epidemiological sampling strategy. The small numbers of participants may account for the lack of statistical associations observed between MES and the independent variables. Recruitment bias is possible because participants were invited to take part by their clinicians on a sequential basis. Blood test results were obtained from recent data recorded in out-patient case notes; it is possible that service users whom have attended for tests may have been invited due to concerns about their physical health and therefore be at a relatively higher risk of MES than those patients with SMI who have not had blood tests recorded recently.

Although this study has a relatively small number of participants, the findings provide sufficient evidence to support the need for intervention studies in this setting and reinforce the requirement to conduct regular MES screening. Future research in Hong Kong should also aim to establish the prevalence of MES in patients with SMI using a larger sample size and a randomised epidemiological sampling strategy.

## Conclusion

The results demonstrate that physical health inequalities in patients with severe mental illness in Hong Kong are similar to those observed in western countries. MES was found to highly prevalent in this population as 35% of the participants (31% men and 39% women) met the IDF diagnostic criteria. The relative risk of metabolic syndrome in this cohort in comparison to the general Hong Kong population is 2.008 (1.737 for males and 2.230 for females). In this study being prescribed first generation antipsychotics and sleep disruption are significant risk factors for MES, whilst eating less than recommended levels of fruits/vegetables and being married are weakly associated with the syndrome. The findings therefore suggest that clinicians in Hong Kong should conduct regular physical health screening for MES irrespective of the type of antipsychotic prescribed, ensure that patients with identified physical comorbidities receive medical treatment promptly and increase the focus of clinical interventions towards physical health promotion strategies.

## Competing interests

Professor Richard Gray has received honoraria and provided consultancy to AstraZeneca, Bristol-Myers Squibb, Jannsen Cilag, Eli Lilly and Co. Otsuka Pharmceutical Europe Ltd, Pfizer, received honoraria from AstraZeneca, Bristol- Myers Squibb, Jannsen Cilag, Eli Lilly and Co. Otsuka Pharmceutical Europe Ltd, Lunbeck, Pfizer, Wyeth and had research funding from AstraZeneca, the Medical Research Council, the National Institute for Mental Health, the Department of Health, Comic Relief. Dan Bressington has received honorarium payments for educational consultancy from Bristol-Myers Squibb, Lundbeck and Jannsen-Cilag. The other authors declare no potential conflicts of interest. This study was not funded by any external body.

## Authors’ contributions

DB was the project lead, designed the study, contributed towards data input, jointly analysed data, interpreted the data analysis and was lead for the writing of the article. JM was the lead for data collection, contributed towards the study design and commented on the final paper. R G provided advice on study design, delivered the training, advised on data analysis and contributed towards the final paper. EC provided advice on study design, advised on data analysis/interpretation and contributed towards the final paper. JP jointly input data, helped interpret the data analysis and contributed towards writing the final paper. AC provided advice on the data analysis strategy, jointly analysed data and contributed towards the final paper. All authors read and approved the final manuscript.

## Pre-publication history

The pre-publication history for this paper can be accessed here:

http://www.biomedcentral.com/1471-244X/13/87/prepub
